# Risk factors for postpartum depression among Chinese women: path model analysis

**DOI:** 10.1186/s12884-017-1320-x

**Published:** 2017-05-02

**Authors:** Shiping Liu, Yan Yan, Xiao Gao, Shiting Xiang, Tingting Sha, Guangyu Zeng, Qiong He

**Affiliations:** Department of Epidemiology and Health Statistics, Central South University Xiangya School of Public Health, Changsha, Hunan China

**Keywords:** Depression, Path analysis, Postpartum, Risk factors

## Abstract

**Background:**

Postpartum depression causes harm to both mothers and infants. The purpose of this study was to find out several potential risk factors, and to identify the intrinsic interrelationships between factors and postpartum depression by constructing a path model. The results of this study may help to control the increasing incidence of maternal postpartum depression.

**Methods:**

The study was based on a sample of mothers from a cross-sectional study which was set up at 4 weeks after a mother had childbirth and was conducted in three streets at Kaifu District of Changsha in Hunan province from January to December 2015. Questionnaires were distributed to subjects who responded to questions concerning factors related to pregnancy, delivery and infants within 4 weeks after childbirth. The Edinburgh Postnatal Depression Scale (EPDS) was used to measure postpartum depression. Chi-square test was used to detect significant differences between non-postpartum depression group and postpartum depression group. A path model was constructed to explore the interrelationships between variables, and to verify the relationships between variables and postpartum depression.

**Results:**

The proportion of maternal postpartum depression was 6.7%. Univariate analysis showed that there were significant differences between non-postpartum depression group and postpartum depression group (all *P*-values <0.05) on the part of maternal age, parity, frequent exposure to mobile phone during pregnancy, gestational hypertensive disorders, fetus number, premature delivery, birth weight, initiation of breastfeeding, mode of feeding, infant illness within 4 weeks after delivery and infant weight at 4 weeks. Path analysis results showed that the final model could be fitted well with sample data (*P* = 0.687, CMIN/DF = 0.824, NFI = 0.992, RFI = 0.982, IFI = 1.002, TLI =1.004, CFI = 1.000 and RMSEA < 0.001). Frequent exposure to mobile phone during pregnancy, maternal age and gestational hypertensive disorders had both direct and indirect effects on postpartum depression. Mode of feeding and infant weight at 4 weeks, which was the most total effect on postpartum depression, had only a direct impact on postpartum depression. Fetus number, premature delivery, initiation of breastfeeding and birth weight had only an indirect influence on postpartum depression.

**Conclusion:**

The findings of this study suggest that constructing a path analysis model could identify potential factors and explore the potential interrelations between factors and postpartum depression. It is an effective way to prevent maternal postpartum depression by taking appropriate intervention measures and carrying out health education for pregnant women.

**Electronic supplementary material:**

The online version of this article (doi:10.1186/s12884-017-1320-x) contains supplementary material, which is available to authorized users.

## Background

Maternal postpartum depression (PPD) is referred to as a constant low mood, with the symptoms of feeling sad, worthless and hopeless, etc, discerned in mothers who have recently gone through their childbirth. It is a common and serious mental disorder which affects 13% to 19% of postnatal women [1].

Postpartum depression is dangerous for both mothers and infants. On the one hand, depression can result in maternal mental disorders [2], infanticide [3], and even suicide [4]. On the other hand, children whose mothers bear the symptoms of postpartum depression have been observed as having greater susceptibility to behavioral and emotional problems [5]. Some studies have identified several possible risk factors associated with maternal postpartum depressive symptoms, including younger maternal age, lower educational level, smoking during pregnancy, history of depression, poor marriage status, poor family economic status, negative life events, lack of social support, antenatal depression and anxiety [1, 6, 7]. However, the risk factors for postpartum depression, though being numerous and complicated, are seldom rendered clear.

Previous studies mostly used the methods of univariate factor and regression analysis to identify the association between risk factors and postpartum depression [8]. Although several variables have no direct effects on postpartum depression, there are possible effects which act as intermediary variables to connect them. A recent study has shown that pre-pregnancy obesity has an indirect effect on postpartum depression through a mediated variable — stress [9]. It indicated that omitting the interrelations between variables could prevent us from finding out potential risk factors for postpartum depression. And their interrelationships cannot be well revealed by traditional analytical methods.

Accordingly, we aimed to identify the interrelationships between factors of postpartum depression and find out several potential risk factors as well as their interconnections by using a path model. It was to reveal how these factors influenced postpartum depression in order to take interventions and, hopefully, help to control the increasing incidence of postpartum depression.

## Methods

### Participants

The study was based on a sample of mothers from a cross-sectional study, which was set up at 4 weeks after a mother had childbirth, and which was conducted in three streets at Kaifu District of Changsha in Hunan province from January to December 2015. This study was approved by ethics review committee of the Institute of Clinical Pharmacology of Central South University (registration number: CTXY-130041-3-2). Eligible participants were those who: 1) delivered from January to December 2015, 2) had a live birth, 3) had no history of mental illnesses or brain diseases, and 4) agreed to participate in this survey and sign an informed consent.

### Model construction

A stress process model, which contains the stressors, mediators and stress outcomes, was applied to this study [10]. Stressors, which can be internal, external, environmental, social, biological or psychological factors, challenge an individual to adapt or modify. Mediators, which can have social or personal resources, attenuate the effects of stressors or change the situations that are producing the stressors. Stress outcomes are psychological or physiological conditions resulting from exposure to stressors, after accounting for the mediators. Stressors can directly result in stress outcomes, or indirectly affect stress outcomes by mediators. In this study, maternal age, fetus number and frequent exposure to mobile phone during pregnancy, were viewed as stressors; gestational hypertensive disorders, delivery-related factors and infant-related factors within 4 weeks after childbirth were regarded as mediators; and postpartum depression symptoms represent stress outcomes.

### Research variables

Maternal postpartum depression was considered to be the primary outcome variable in this study. The risk factors for postpartum depression, according to chronological order, contained the following three aspects: pregnancy-related factors, delivery-related factors and infant-related factors within 4 weeks after childbirth. The pregnancy-related factors were maternal age (<25, 25 ~ 29 or ≥30), educational level (≤junior school, senior school or ≥ college), average monthly family income (≤2000, 2001 ~ 5000 or >5000), parity (primiparous or multiparous), history of cesarean section (yes or no), gestational hypertensive disorders (yes or no), gestational diabetes mellitus (yes or no), passive smoking during pregnancy (yes or no) and frequent exposure to mobile phone during pregnancy which meant that women exposed to mobile phone for more than 15 min at one time during pregnancy (yes or no). The delivery-related factors included low birth weight which meant that *z*-score of infant birth weight was below -2 standard deviations (SD) for weight-for-age [11, 12] (yes or no), infant gender (male or female), mode of delivery (vaginal delivery or cesarean delivery), fetus number (singleton or multiple), premature delivery described as a living birth before 37 weeks gestation [13] (yes or no), initiation of breastfeeding (≤2 h or >2 h) and number of family inhabitants (≤3 or >3). The infant-related factors included low weight at 4 weeks of age which meant that *z*-score of infant weight at 1 month was below -2SD for weight-for-age [11, 12] (yes or no), mode of feeding (exclusive breastfeeding, mixed feeding or formula feeding) and infant illness from childbirth to 4 weeks after delivery (yes or no).

### Measure

The Edinburgh Postnatal Depression Scale (EPDS) was used extensively to evaluate maternal depression at 4 weeks after childbirth, which contained a total of 10 items. Each item was split into 4 grades (0 to 3), according to the presence and intensity of postpartum depression, and the total scores ranged from 0 to 30 points [14]. The higher the scores were, the severer the symptom of postpartum depression would be. The cut-off point was defined as more than 10, which screened postpartum depressive symptoms [14]. The Chinese version of EPDS has been demonstrated a satisfactory psychometric properties [15]. The Cronbach’s alpha coefficient of EPDS was 0.856 in our study.

### Data collection and procedure

This study was conducted in three streets at Kaifu District of Changsha in Hunan province. The data collected in this investigation, which contained demographic characteristics and relevant information of mothers and infants, were used for this analysis. Additional file 1 listed the questionnaire. Trained investigators, by means of the home visit, distributed questionnaires to mothers at 4 postpartum weeks and responded to questions concerning factors related to pregnancy, delivery and infants within 4 weeks after childbirth. When related information about mothers or infants was missing or inappropriate, maternal and child healthcare handbooks which were held directly by participants and contained some information about maternal delivery and infant growth and development, were used to extract or check it. Trained investigators were given unified survey standards before conducting the formal investigation. At the same time, pre-investigation was conducted to improve the content of questionnaires and to ensure the objectivity and accuracy of our data.

In total, 976 postpartum questionnaires were gathered, among which 94 were not complete, so there were in the end 882 eligible samples.

### Data analysis

The Statistical Package for Social Science (SPSS), software version 18.0 was used to analyze data. Chi-square test was used to detect significant differences between non- postpartum depression group and postpartum depression group. Path analysis was used to explore the interrelations between variables, and to verify the associations between variables and postpartum depression. The path model was conducted in chronological order. It included three time periods: 1) pregnancy, 2) the time at childbirth, and 3) 4 weeks after delivery. The initial path model was established by Analysis of Moment Structures (AMOS), software version 18.0, which was utilized to draw the path diagram. The modified model was conducted appropriately according to modification indices and was timed to obtain the final path model. Standardized regression weights were used to represent path coefficients between variables with *P*-values less than 0.05. The overall fitting model was considered to be acceptable as following values: ratio of likelihood-ratio *χ* ^2^ values to degrees of freedom values (CMIN/DF) less than two times, goodness-of-fit index (GFI), adjusted goodness-of-fit index (AGFI), normed fit index (NFI), relative fit index (RFI), incremental fit index (IFI), Tacker-Lewis fit index (TLI), comparative fit index (CFI) of all values greater than or equal to 0.900, and root mean square error of approximation (RMSEA) values lower than or equal to 0.080 [16]. All *P*-values less than 0.05 were considered as statistically significant. Eventually, only the significant paths could be included in the model.

## Results

In this study, 882 eligible participants delivered with live births. The average of maternal age, infant birth weight and infant weight at 4 weeks were 29.9(4.0), 3.3(0.5) and 4.5(0.7), respectively. The proportion of maternal postpartum depression was 6.7%.

Univariate analysis showed that there were significant differences between non-postpartum depression group and postpartum depression group (all *P*-values <0.05) on the part of maternal age, parity, frequent exposure to mobile phone during pregnancy, gestational hypertensive disorders, fetus number, premature delivery, birth weight, initiation of breastfeeding, mode of feeding, infant illness within 4 weeks after delivery and infant weight at 4 weeks (see Table 1).

**Table 1:** Comparisons of basic characteristics between groups of non-PPD and PPD

Basic characteristics	Non- PPD	PPD	Chi-square test	*P* value
	*n* (%)	*n* (%)		
Maternal age(year)				
< 25	42(5.1)	12(20.3)	22.342	<0.001
25 ~ 29	378(45.9)	24(40.7)		
≥ 30	403(49.0)	23(39.0)		
Birth weight				
< -2SD	12(1.5)	11(18.6)	57.437	<0.001
≥ -2SD	811(98.5)	48(81.4)		
Educational level				
≤ junior school	26(3.2)	2(3.4)	0.972	0.615
Senior school	106(12.9)	5(8.5)		
≥ college	691(84.0)	52(88.1)		
Average monthly family income(RMB)				
≤ 2000	23(2.8)	3(5.1)	1.890	0.389
2001 ~ 5000	448(54.4)	35(59.3)		
> 5000	352(42.8)	21(35.6)		
Number of family inhabitants				
≤ 3	172(20.9)	17(28.8)	2.048	0.152
> 3	651(79.1)	42(71.2)		
Mode of delivery				
Vaginal delivery	496(60.3)	34(57.6)	0.160	0.689
Cesarean delivery	327(39.7)	25(42.4)		
Gestational hypertensive disorders				
No	817(99.3)	55(93.2)	12.988	<0.001
Yes	6(0.7)	4(6.8)		
Gestational diabetes mellitus				
No	786(95.5)	54(91.5)	1.145	0.285
Yes	37(4.5)	5(8.5)		
Fetus number				
Singleton	807(98.1)	50(84.7)	30.745	<0.001
Multiple	16(1.9)	9(15.3)		
Premature delivery				
No	791(96.1)	48(81.4)	22.764	<0.001
Yes	32(3.9)	11(18.6)		
Initiation of breastfeeding				
≤ 2 h	558(67.8)	26(44.1)	13.861	<0.001
> 2 h	265(32.2)	33(55.9)		
Passive smoking during pregnancy				
No	744(90.4)	53(89.8)	0.021	0.698
Yes	79(9.6)	6(10.2)		
Frequent exposure to mobile phone during pregnancy				
No	554(67.3)	26(44.1)	13.213	<0.001
Yes	269(32.7)	33(55.9)		
History of cesarean section				
No	777(94.4)	58(98.3)	0.973	0.324
Yes	46(5.6)	1(1.7)		
Parity				
Primiparous	584(71.0)	51(86.4)	6.544	0.011
Multiparous	239(29.0)	8(13.6)		
Infant gender				
Male	430(52.2)	32(54.2)	0.087	0.768
Female	393(47.8)	27(45.8)		
Infant weight at 4 weeks				
< -2SD	10(1.2)	14(23.7)	97.085	<0.001
≥ -2SD	813(98.8)	45(76.3)		
Mode of feeding				
Exclusive breastfeeding	681(82.7)	36(61.0)	25.598	<0.001
Mixed feeding	131(15.9)	18(30.5)		
Formula feeding	11(1.3)	5(8.5)		
Infant illness within 4 weeks after childbirth				
No	767(93.2)	46(78.0)	15.658	<0.001
Yes	56(6.8)	13(22.0)		

*Abbreviation*: *PPD* Postpartum Depression, *SD* Standard Deviation

**Fig. 1:**
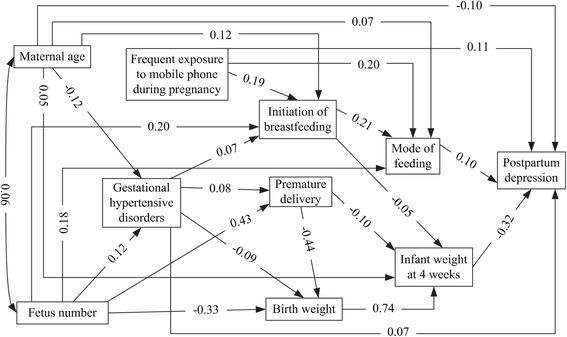
Path model of relationships between variables and maternal PPD among Chinese women

The model that only included significant path coefficients was presented in Fig. 1, and the estimates of standardized direct, indirect and total effects on postpartum depression were shown in Table 2. Frequent exposure to mobile phone during pregnancy could not only directly affect postpartum depression (0.105), but also indirectly influence postpartum depression through initiation of breastfeeding, infant weight at 4 weeks and mode of feeding (0.028). Additionally, two variables, that is, maternal age and gestational hypertensive disorders, had both direct and indirect effects on postpartum depression. The total effect coefficients were -0.115 and 0.101 respectively. Infant weight at 4 weeks—the most total effect on postpartum depression, and mode of feeding had only a direct impact on postpartum depression, while they functioned as important mediators between other variables and postpartum depression. The direct effect coefficients of them were -0.319 and 0.102 respectively. Fetus number, premature delivery, initiation of breastfeeding and birth weight had only an indirect effect on postpartum depression. The indirect effect coefficients of them were 0.173, 0.135, 0.038 and -0.235 respectively.

**Table 2:** Summary of the direct, indirect and total effects on postpartum depression

Variables	Effects		
	Direct	Indirect	Total
Maternal age	-0.100	-0.015	-0.115
Gestational hypertensive disorders	0.066	0.035	0.101
Frequent exposure to mobile phone during pregnancy	0.105	0.028	0.133
Fetus number	0.000	0.173	0.173
Premature delivery	0.000	0.135	0.135
Birth weight	0.000	-0.235	-0.235
Initiation of breastfeeding	0.000	0.038	0.038
Infant weight at 4 weeks	-0.319	0.000	-0.319
Mode of feeding	0.102	0.000	0.102

Infant illness and parity had neither direct nor indirect impact on postpartum depression in the final path model. Although bearing statistical correlations with postpartum depression in the univariate factor analysis, they were excluded from the model.

The results of fitting indices of this path model indicated that the hypothetical model could be fitted well with sample data (*P* = 0.687, CMIN/DF = 0.824, NFI = 0.992, RFI = 0.982, IFI = 1.002, TLI =1.004, CFI = 1.000 and RMSEA < 0.001).

## Discussion

Our results indicated that maternal postpartum depression should be studied synthetically without neglecting especially any potential factors. It was therefore necessary to construct a path analysis model so as to identify several potential factors and better explore how different factors influenced postpartum depression.

Interestingly, we found that pregnant women’s frequent exposure to mobile phone was not only directly related to postpartum depression. Meanwhile, mothers who were frequent users of mobile phones were more inclined to delay their initiations of breastfeeding and feed infants with formula rather than breast milk, and their infant weights were lighter at 4 weeks. Thus, these women might be affected by postpartum depression in the form of indirect pathway, which was one of the important findings in this study. As exposure to mobile phone was an important behavior factor in leading to postpartum depression, pregnant women should have a moderate use of mobile phones. A previous study noted that there was no association between maternal exposure to mobile phone and unfavorable outcomes [17]. In this study, women of 15 and 30 weeks’ pregnancy were inquired about two questions: “How often do you talk on mobile phone” and “Do you talk on mobile phone for more than 15 min at one time”, by which maternal exposure to mobile phone was examined. Nevertheless, in our study, mothers were asked to answer whether they frequently use the mobile phone during pregnancy in a retrospective manner, and the corresponding answers were yes/no. Further research is needed to confirm and specify this association.

On the one hand, younger maternal age led to a higher level of postpartum depression in the form of direct pathway, which was in line with previous research [18]. On the other hand, maternal age showed indirect pathway to postpartum depression by influencing premature delivery, birth weight, initiation of breastfeeding, infant weight at 4 weeks and mode of feeding. Since maternal age is a fixed factor, other changeable factors acting as mediators such as gestational hypertensive disorders and premature delivery, might serve as primary intervention factors to prevent the incidence of postpartum depression. Moreover, gestational hypertensive disorders could be directly associated with postpartum depression, which was consistent with previous studies as well [19, 20]. We also found that gestational hypertensive disorders could indirectly affect postpartum depression through premature delivery, birth weight, initiation of breastfeeding, infant weight at 4 weeks and mode of feeding. This disorder resulted in a higher risk of unfavorable pregnancy outcomes which were disadvantageous for the well-being of both mothers and children. They might be susceptible to worry and anxiety. It is therefore essential for pregnant women to monitor blood pressure regularly and keep it steady. This may help improve pregnancy outcomes and relieve the predisposition of maternal postpartum depression.

Our research showed that two variables had only direct effect on postpartum depression and played the important mediating roles between other variables and postpartum depression. We found that women who fed their infants with formula or a mixture of breast milk and formula were more susceptible to postpartum depression than women who only breasted. This was consistent with previous studies [21–23]. It suggests that formula feeding or a mixture of breast milk and formula feeding is not conducive to mother-infant attachment, and the best way to reduce the risk of maternal postpartum depression is to adopt exclusive breastfeeding. It is therefore essential for medical personnel to increase women’s prenatal and postnatal health awareness by encouraging breastfeeding. Another variable that was directly linked to maternal postpartum depression was infant weight at 4 weeks, which was the most total effect on postpartum depression. A previous study has shown that significantly elevated risk factors for postpartum depression include concerns of infant weight gain [24]. For women whose infant weight gain is insufficient, they are more inclined to be concerned about the health and development of their babies compared with women whose infant has normal weight.

Unexpectedly, we found that four variables affected postpartum depression only in an indirect pathway. Although not directly connected with postpartum depression, they could indirectly affect postpartum depression by acting on other factors, thus serving as important potential factors. Our findings showed that multiple pregnancies indirectly affected postpartum depression by directly influencing gestational hypertensive disorders, premature delivery, birth weight, initiation of breastfeeding and mode of feeding, as well as by indirectly impacting infant weight at 4 weeks. Thus, multiple pregnant women might be more susceptible to postpartum depression than singleton mothers. This was consistent with previous studies [25]. As mentioned in our results, premature infants usually had low birth weight and poor physique. And it was certainly more possible for them to contract disease than non-premature infants. Therefore, women with premature delivery had a higher risk of postpartum depression [26]. In addition, infants with low birth weight had lighter weight at 4 weeks of age, which were only indirectly linked to maternal postpartum depression, because their mothers are more worried about their growth or even undergo an excessively strong anxiety [27]. Furthermore, compared to infants who were breastfed within 2 h of birth, infants who were breastfed more than 2 h after birth were more likely to be fed with formula and had lower weights at 4 weeks. It, therefore, increased the likelihood of maternal postpartum depression. This was in line with previous studies [21, 28]. It is thus necessary to promote prenatal care and initiate breastfeeding as early as possible. Apart from prenatal care, postpartum care will also help to decrease the rate of postpartum depression, and keep mothers in a good mood.

Previous studies on postpartum depression centered on patients in the hospital, while our study was concerned with people in the community, which could reduce selection bias. Though, recall bias inevitably existed in our study, because our investigation focused on 4-week period after childbirth and used retrospective method. However, details of relevant information were collected by means of combining field investigation with maternal and child healthcare handbooks, to ensure that our data were as complete and correct as possible. Despite that our study didn’t consider the impact of spouse or family support, depression during pregnancy and other negative life events on postpartum depression, it concentrated on identifying further risk factors for postpartum depression from the perspectives of pregnancy, childbirth and infant factors. Although the whole model contributed less to the variance of postpartum depression, it disclosed potential risk factors and would help to control alterable factors and reduce the incidence of postpartum depression.

## Conclusions

Constructing a path analysis model could identify potential factors and explore the potential interrelations between factors and postpartum depression. It is an effective way to prevent maternal postpartum depression by taking appropriate intervention measures and carrying out health education for pregnant women, such as the importance of an early initiation of breastfeeding, a steady blood pressure and less exposure to mobile phone during pregnancy. Hopefully, this may help decrease the risk of premature delivery and low birth weight and improve the health condition of both mothers and infants.

## Additional file

Additional file 1: Questionnaire. (DOCX 28 kb)

## Abbreviations

AGFI: Adjusted goodness-of-fit index
AMOS: Analysis of moment structures
CFI: Comparative fit index
CMIN/DF: Minimum value of the discrepancy function divided by degrees of freedom
EPDS: Edinburgh postnatal depression scale
GFI: Goodness-of-fit index
IFI: Incremental fit index
NFI: Normed fit index
PPD: Postpartum depression
RFI: Relative fit index
RMSEA: Root mean square error of approximation
SD: Standard deviations
SPSS: Statistical package for social science
TLI: Tacker-Lewis fit index
