# Supplementary material for: Risk factors for postpartum depression among Chinese women: path model analysis
Source: BMC Pregnancy Childbirth. 2017 May 2;17:133. doi: 10.1186/s12884-017-1320-x (PMC5414210; doi:10.1186/s12884-017-1320-x)
Supplement: Additional file 1: — Questionnaire. (DOCX 28 kb) [file 12884_2017_1320_MOESM1_ESM.docx]

**Maternal and Child Health Information Collection at Kaifu District of Changsha in 2015**

**Pregnancy-related information**

1. District

2. Street

3. Maternal name

4. Maternal age

5. Maternal educational level

(1) ≤Primary School (2) Junior School (3) Senior School (4) College (5) ≥Master (6) Other (please specify)

6. Average monthly family income (yuan/month)

(1) ≤2000 (2) 2001~5000 (3) 5001~10000 (4) 10001~15000 (5) >15000

7. Do you have a history of cesarean section before this pregnancy?

(1) No (2) Yes

8. Do you have a history of mental illnesses before this pregnancy?

(1) No (2) Yes

9. Do you have a history of brain diseases before this pregnancy?

(1) No (2) Yes

10. What was your parity?

11. Did you frequently use a mobile phone for more than 15 minutes at one time during pregnancy?

(1) No (2) Yes

12. Which of the following options did you have during pregnancy (you can make multiple choices)?

(1) None (2) Multiple gestation (3) Gestational hypertensive disorders (4) Placenta previa (5) Abnormal amniotic fluid (6) Fetal growth restriction (7) Fetal abnormalities (8) Gestational diabetes mellitus (9) Delayed pregnancy (10) Post-term pregnancy (11) Threatened preterm labor (12) Late threatened abortion (13) Stillbirth (14) Macrosomia (15) Moderate anemia (16) ABO hemolytic (17) Pregnancy liver injury (18) Other (please specify)

**Delivery-related factors information in this pregnancy**

1. When did you give birth?

2. What was your gestational age?

3. What was your infant’s gender?

4. What was weight of your infant’s birth? Kg

5. What was your mode of delivery?

(1) Spontaneous vaginal delivery (with side-cut) (2) Spontaneous vaginal delivery (without side-cut) (3) Cesarean delivery (4) Assisted delivery

6. Did you give a live birth?

(1) Yes (2) No

7. How long did your infant begin to initiate breastfeeding after birth?

(1) ≤2 hours (2) >2 hours

8. How many people did your home inhabit?

**Infant-related factors within 4 weeks after childbirth information**

1. Which mode do you feed your infant?

(1) Exclusive breastfeeding (2) Mixed feeding (3) Formula feeding

2. What’s weight of your infant at 4 weeks of age? Kg

3. Which of the following options does your infant have from birth to 4 weeks (you can make multiple choices)?

(1) None (2) Cold (3) Diarrhea (4) Bronchitis (5) Pneumonia (6) Asthma (7) Rickets (8) Anemia (9) Debility child (10) Other (please specify)

**Edinburgh Postnatal Depression Scale (Evaluating maternal depression at 4 weeks after childbirth)**

Please make responses to the following options:

1. I have been able to laugh and see the funny side of things

(1) As much as I always could (2) Not quite so much now (3) Definitely not so much now (4) Not at all

2. I have looked forward with enjoyment to things

(1) As much as I ever did (2) Rather less than I used to (3) Definitely less than I used to (4) Hardly at all

3. I have blamed myself unnecessarily when things went wrong

(1) No, never (2) Not very often (3) Yes, some of the time (4) Yes, most of the time

4. I have been anxious or worried for no good reason

(1) No, not at all (2) Hardly ever (3) Yes, sometimes (4) Yes, very often

5. I have felt scared or panicky for no very good reason

(1) No, not at all (2) No, not much (3) Yes, sometimes (4) Yes, quite a lot

6. Things have been getting on top of me

(1) No, I have been coping as well as ever (2) No, most of the time I have coped quite well (3) Yes, sometimes I haven’t been coping as well as usual (4) Yes, most of the time I haven’t been able to cope at all

7. I have been so unhappy that I have had difficulty sleeping

(1) No, not at all (2) Not very often (3) Yes, sometimes (4) Yes, most of the time

8. I have felt sad or miserable

(1) No, never (2) Only occasionally (3) Yes, quite often (4) Yes, most of the time

9. I have been so unhappy that I have been crying

(1) No, never (2) Only occasionally (3) Yes, quite often (4) Yes, most of the time

10. The thought of harming myself has occurred to me

(1) Never (2) Hardly ever (3) Sometimes (4) Yes, quite often
